# Cough and Its Treatment

**Published:** 1912-01

**Authors:** Minnie M. Hopkins


### Cough and Its Treatment.

Cough is not in and of itself a disease, but a symptom of one. There is the grip cough, the measles cough, the asthmatic cough, the croupy cough, the heart and stomach cough. I would like to be systematic and outline the remedies for each, but the symptomatology differs so widely that I must confine myself to the group of remedies that has been most helpful to me the past winter. In the epidemic of whooping cough through which we have just passed, and in the form of grip which has attacked many of the grown-ups, I have more often found aid in iodized calcium than in any other remedy. Some years ago I heard a lecture by Dr. Beebe, of Chicago, on this remedy in croup. It has long been the first remedy thought of in croup, and my experience with it there led me to think of it in connection with the paroxysms of whooping cough; but when I came to search for confirmation in the books I could not find it, so shall have to give you clinical experience only. Abbott makes a combination of iodine and calcium which he says is 15 per cent available iodine and 85 per cent of calcium and it does good work, and he gives clear indications for its use. The indications for the use of calcium iodized are:

1. Severe spasmodic cough; the face becoming almost purple and the patient seems to be about to lose his breath.

2. The muscles seem to be tense and rigid, and when the paroxysm is over the child is exhausted, the forehead bathed in perspiration.

3. The child dreads another attack and wants to be held close.

I have seen calcium iodized within a short time so modify the cough that, while it did not entirely disappear, it was so mild that the child did not much mind, and the dread whoop or spasmodic action of the breathing muscles was entirely absent.

Case 1.—Small boy; flaxen hair; chubby build; had frequent paroxysms of cough, and he said, "I have whistles inside of me," so modified in a week that there were no night vigils, cough became very slight and there was no whoop.

Case 2.—Little girl, aged 4, short and stumpy in build, light hair and eyes, coughs until she cries and sometimes loses her breath. I saw the case only four times, and from the first there was marked relief.

Now is the time to make preparation for the fall colds. Calcidin is one of the indispensable remedies for the ailments of the respiratory tract. Unlike most so-called "cough remedies," it not merely relieves—it goes to the seat of the trouble. It should, of course, be used in association with other agents. Thus, in whooping cough, most physicians use it in association or alternation with calcium sulphide, which has a peculiar potency in cutting short the disease; and with atropine, which modifies the severity of the paroxysms. Other remedies which are useful in this disease are monobromated camphor, lobelin, cicutine and quinine hydroferrocyanide. These are variously combined in two excellent formulas of our list. "Whooping Cough" (Cushman) and "Whooping Cough (Abbott)." The first of these sell at 13 cents for 100, 53 cents for 500, $1.00 for 1000; the second sells for 16 cents for 100, 65 cents for 500, and $1.25 for 1000.

Coughs are troublesome things when they attack the baby. The doctor is afraid to give opium and its salts to control them—and he should be. There is a better way. If there is fever, as there usually is, administer aconitine in small, repeated doses, according to the Shaller rule. Anoint the chest well with our antiseptic oil, applying warm, and covering with hot flannel. Then control the cough with Anodyne for Infants (Waugh), using small doses of emetin if cough is tight, or sanguinarine if the chest is filled with coarse rales, showing an accumulation of secretion. These granules can be dissolved in hot water, and the solution sweetened with honey, or orange syrup. Calcidin, in one-third-grain tablets, completes the medication.

I should like to tell you about a lot of splendid cough combinations suitable for adults, but space is limited—I can only remind you of Coryza, No. 1 and No. 2, to break up the beginning "colds in the head"; of Cough (Abbott), for the beginning tight cold "on the chest"—with some fever; of Cough (Blackman) for a similar purpose, but containing tartar emetic and pilocarpine, and insuring energetic action; of cough nervous, whose name indicates its field. Doubtless you are familiar with all of these, and need to be reminded to add a goodly supply to your next order.

Don't forget Calcidin. You will be using a lot of this in the next two or three months. It is now obtainable in one-third-grain, one-grain and two-grain tablets, and in five-grain capsules, and in bulk. Better get a variety of these forms. Cheaper and more convenient.—Ed.—Dr. Minnie M. Hopkins, in the *Clinique*.

---

The dew was falling fast,
  The stars began to blink,
I heard a voice that said,
  "Drink, pretty creature, drink!"
But "Baa!" the lamb replied,
  "I will not take one sup
Till you provide an in-
  Dividual drinking cup."

---

### When I Am Dead.

When I am dead, if man can say
"He helped the world upon its way,
With all his faults of word or deed
Mankind did have some little need
Of what he gave"—then in my grave
No greater honor shall I crave.
If they can say—if they but can—
"He did his best, he played the man,
His ways were straight; his soul was clean;
His failings not unkind nor mean,
He loved his fellow men, and tried
To help them"—I'll be satisfied.

